# Macronutrient intake inadequacy and associated factors among school adolescent girls in Meshenti, Northwest Ethiopia, 2020: 24-h recall

**DOI:** 10.1017/S1368980025100736

**Published:** 2025-08-05

**Authors:** Birtukan Gizachew Ayal, Yeshalem Mulugeta Demilew, Hunegnaw Almaw Derseh

**Affiliations:** 1 School of Public Health, College of Health Sciences, Woldia University, P.O. Box 400, Woldia, Ethiopia; 2 Department of Nutrition and Dietetics, School of Public Health, College of Medicine and Health Sciences, Bahir Dar University, P.O. Box 79, Bahir Dar, Ethiopia

**Keywords:** Adolescent girls, Macronutrient intake inadequacy, Ethiopia

## Abstract

**Objective:**

This study assessed macronutrient intake and associated factors among school adolescent girls in Meshenti, Northwest Ethiopia, 2020.

**Design:**

A cross-sectional study was conducted from 7 to 23 February 2020, among 401 randomly selected adolescent girls. Macronutrient intake was assessed using a 24-h dietary recall with portion size estimation. Nutrient data were analysed with Elizabeth Stewart Hands and Associates FOOD PROCESSOR software and compared with WHO/FAO recommendations – 2200 kcal for energy and 34–46 g for protein. Factors associated with inadequate macronutrient intake were identified using multivariable logistic regression.

**Setting:**

The study was conducted in an institutional setting.

**Participants:**

This study was conducted among school adolescent girls.

**Results:**

The median (IQR) energy intake was 2040·23 (1648·24–2744·51), and the mean (sd) protein intake was 63·88 (20·99). About 57·6 % (95 % CI: 52·9, 62·8 %) had inadequate energy intake, and 18·5 % (95 % CI: 14·7, 22·2 %) had inadequate protein intake. Inadequate energy intake was associated with dietary diversity (AOR = 4·31, 95 % CI: 2·20, 8·47), knowledge (AOR = 2·10, 95 % CI: 1·34, 3·28) and meal frequency (AOR = 2·5, 95 % CI: 1·06, 5·95). Factors linked to inadequate protein intake included early adolescence (AOR = 1·89, 95 % CI: 1·08, 3·31), residency (AOR = 0·27, 95 % CI: 0·15, 0·48), dietary diversity (AOR = 3·28, 95 % CI: 1·08, 9·98), knowledge (AOR = 1·82, 95 % CI: 1·04, 3·19) and meal frequency (AOR = 2·94, 95 % CI: 1·35, 6·37).

**Conclusion:**

This study revealed high inadequate energy and protein intake. Contributing factors included dietary diversity, knowledge and meal frequency, with age and residence affecting protein intake. Emphasis is needed on early adolescent girls’ nutrition education.

## Introduction

The WHO defines adolescence as any person between the ages of 10 and 19^(1)^. The world is home to 1·3 billion adolescents, making up 16 % of the world’s population, and the greatest proportions (23 %) of the sub-Saharan African population are in this age group^(2)^.

Adolescence represents a key stage of physical development, second only to infancy, characterised by accelerated growth and changes in the body. During this time – particularly with the onset of menstruation or pregnancy – adolescent girls experience increased nutritional needs, making them especially vulnerable to nutrient deficiencies and malnutrition^(3)^.

Taking into consideration for reduction of chronic diseases while providing intakes of essential nutrients, the WHO, Institute of Medicine (IOM) and FAO recommended for adolescent girls daily intake of 130 g carbohydrate, 34 g protein for age 10–13 years and 46 g protein for age 14–19 years and 2200 kcal energy. The total fat recommended for adolescent girls should not be more than 35 % and not less than 25 % of total calories^(4,5)^.

Inadequate nutrition among adolescent girls is linked to negative maternal health outcomes and complications during childbirth. It can also impair cognitive development and is believed to significantly lower human productivity by 10–15 %, as well as reduce the gross domestic product by as much as 10 % in developing nations. Deaths in children due to malnutrition are also strongly associated with poor nutrition of adolescent girls, which later become malnourished mothers^(1,6–8)^.

Globally, adolescent girls often fail to meet the recommended energy intake levels^(9)^. Despite an overall sufficient intake, protein consumption frequently remains inadequate due to poor dietary quality. On the other hand, carbohydrate intake tends to be sufficient or even excessive among this group. These imbalanced nutrient patterns, along with other contributing factors, have made malnutrition a significant global concern affecting both the health of adolescent girls and future generations^(10)^. In 2015 alone, approximately 1·2 million adolescents died worldwide, with over two-thirds of these deaths occurring in low- and middle-income regions – particularly in Africa (45 %) and Southeast Asia (26 %)^(11)^.

In the world, around 34 deaths per 100 000 adolescents are attributed to malnutrition, and protein-energy malnutrition is among the top 10 causes of these deaths, accounting for 225 906 deaths in 2013^(10)^. In developing countries, energy and macronutrient intake among adolescent girls in general falls below the recommended intake^(12)^. In these regions, inadequate energy and macronutrient intake in adolescent girls was mainly related to psycho-social, socio-demographic or economic characteristics, lifestyle and eating behaviour factors, though these factors were not assessed well in the Ethiopian context^(1,3,13)^.

In Ethiopia, the dual burden of malnutrition in adolescent girls is a current public health problem that is linked to dietary and psycho-social behaviours^(14,15)^. Moreover, few dietary assessment studies in the country also revealed suboptimal energy and macronutrient intake of adolescent girls compared with standard recommendations by WHO/FAO or IOM^(16,17)^. As diet is a risk factor for malnutrition, poor pregnancy outcomes and chronic diseases, it is clear that understanding the dietary macronutrient intake of adolescent girls will be essential to promote better health and nutrition for this group and their future children. However, adolescents have been absent from health plans for decades^(7)^. Moreover, quantitative dietary studies in adolescent girls are scarce in Ethiopia. Pieces of literature mainly focus on school children (aged 6–10 years)^(18)^, include combined data for boys and girls and are only descriptive^(16,17)^. This study looks principally at the dietary intake of macronutrients, and it estimated inadequate energy and protein intake as well as associated factors among school adolescent girls in Meshenti town of Bahir Dar City Administration, Northwest Ethiopia. This would allow for more informed policy and programme development targeted towards this vulnerable group.

## Methods and materials

### Study area

This study was carried out in Meshenti, a rural town within the Bahir Dar City Administration, in February 2020. Located approximately 12 km south of Bahir Dar, the town lies in the Amhara region of Northwest Ethiopia.

Meshenti is home to two public schools – one primary and one secondary. During the study period, a total of 3675 students were enrolled in these schools, with 50 % of them being female. Of these, 1430 were adolescent girls, including 831 at the primary level and 599 at the secondary level, beginning from grade four. No adolescent girls were enrolled in grades 1 through 3^(19)^. The students in the schools came from both the urban Meshenti 01 kebele and surrounding rural areas of Bahir Dar City. The primary economic activities in the region include agriculture in rural areas and trade in urban areas. Common agricultural products include maize, millet, teff, barley, legumes, fruits like mango and banana and coffee. Livestock such as cattle, chickens, sheep and goats are also raised in the area.

### Study design and period

A cross-sectional study was carried out in schools involving 401 adolescent girls from 7 to 23 February 2020.

### Source population and study population

The source population consisted of all adolescent girls enrolled in schools in Meshenti town, while the study population included those attending school during the study period.

#### Sample size determination and sampling technique

#### Sample size determination

The sample size was calculated using a single population proportion, based on the following assumptions: an expected 50 % prevalence of inadequate energy and protein intake among adolescent girls, a 95 % confidence level and a 5 % margin of error. Additionally, a 10 % non-response rate was factored in, resulting in a final sample size of 422.

#### Sampling procedure

**Fig. 1:**
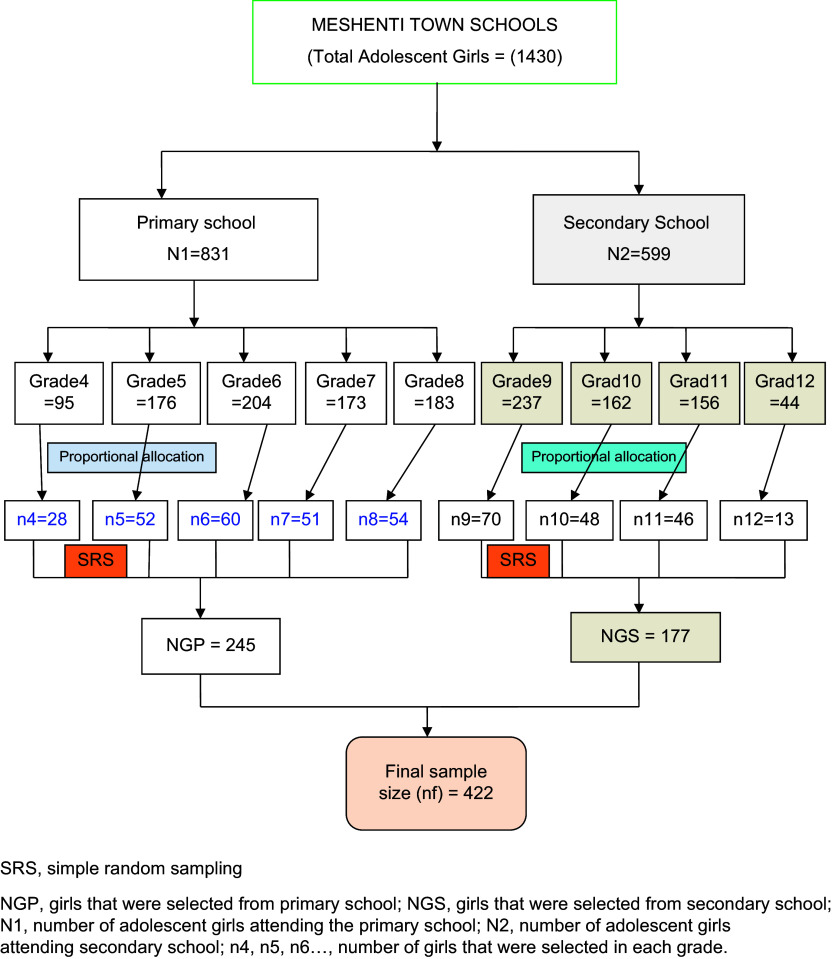
Schematic presentation of the sampling procedure among school adolescent girls in Meshenti Town, Ethiopia, 2020.

Initially, a list of students from both the primary and secondary schools in the town was obtained from the school registrar’s office, including their names, ages, grades and addresses. Adolescent girls were then identified from this list, which contained 1430 girls (831 from primary and 559 from secondary). The list was organised by their identification numbers, which served as the sampling frame. The required sample size of 422 girls was then proportionally allocated based on the number of girls in each school. Ultimately, 245 girls from the primary school and 177 from the secondary school were selected through computer-generated simple random sampling, with the sample distribution proportional to the number of girls in each school and grade (Fig. 1).

#### Data collection tools and procedures

Data were gathered using structured questionnaires administered by an interviewer. The questionnaire covered various aspects, including socio-demographic and economic factors, dietary habits, household food security, knowledge levels, media exposure, peer influence on eating behaviours, body image perceptions and medical conditions, all based on a review of relevant literature^(2,3,20–23)^. Once the study participants were chosen from the schools, their household addresses were obtained from the school’s records. Data collectors then visited the homes of the adolescent girls to conduct the interviews. A team of six public health nutritionists collected data from both the mothers (caregivers) and the adolescent girls.

#### Measurements

#### Dietary assessment

A modified and validated interactive four-pass 24-h dietary recall questionnaire, specifically designed for use in developing countries^(21)^, was employed to estimate portion sizes and evaluate the energy and macronutrient intake from foods and beverages consumed by adolescent girls.

Prior to the actual data collection, a market survey and observation of twenty-one households in the study area were conducted to gather information on commonly consumed foods, cooking methods and the types of serving utensils used. Photographs of the utensils and typical food portions served in one meal were taken during the surveillance. These utensils were then purchased from the market. Following this, each utensil and portion was photographed and assigned a specific code. The utensils used for serving food were standardised with food portions and water using a measuring cylinder and a digital food portion weighing scale (TANITA). The data were reported in millilitres and grams, with 100 ml being considered equivalent to 100 g for beverages.

Interviews were conducted with adolescent girls and their mothers (caregivers). Photographs of household utensils (such as spoons, ladles, cups and glasses), food portions and food models were utilised to aid participants in recalling and determining the portion sizes of the foods consumed. In addition, a list of commonly consumed staple foods in the study area was compiled and read to the participants after the dietary recall, helping them remember any items they might have forgotten.

The amounts of food consumed were estimated using household measurements, local estimations (such as Efign, which represents two hands of an average adult, and Lat, representing one hand of an average adult), as well as by number (for items like oranges, bananas, lemons, mangoes, guavas, boiled potatoes and boiled eggs) and by pieces (for foods like Injera and bread). Foods measured by number were categorised as large, medium or small. Participants were asked to identify the utensil they used from a photographic reference and estimate the portion size based on the equipment. For purchased items such as pasta, biscuits and beverages (e.g. soft drinks), the brand name was recorded along with the quantity consumed, and these items were bought from the market to analyse their nutritional content based on the label. For mixed dishes, the nutrient values were determined from their recipes.

In addition to evaluating nutrient intake, the 24-h recall data were used to calculate the dietary diversity score (DDS) for the adolescents. Dietary diversity was measured using a standardised tool recommended by the FAO, which is typically used to assess women’s dietary diversity. The foods consumed within the 24-h period were classified into ten food groups based on their nutrient content: grains (including white roots, tubers and plantains), pulses (such as beans, peas and lentils), nuts and seeds, dairy products, meat (including poultry and fish), eggs, dark green leafy vegetables, vitamin A-rich fruits and vegetables, other vegetables and fruits. A DDS was then generated as a summary of dietary diversity. Adolescent girls who consumed five or more food groups were classified as having a high DDS, while those who consumed fewer than five food groups were categorised as having a low DDS^(22)^.

##### The wealth index of the households

The determination was made using principal component analysis, which took into account factors such as latrine availability, water source, household assets, livestock, agricultural land ownership and crop production, as adapted from the 2016 Ethiopian Demographic and Health Survey^(20)^.

##### Knowledge

Questions were designed to assess knowledge regarding the sources of nutrients, the benefits of nutrients and the nutritional needs of adolescent girls. Those who answered the knowledge assessment questions correctly and scored above the mean were considered to have adequate knowledge, while those who scored below the mean were deemed to have insufficient knowledge^(23)^.

##### Peer pressure influence

The Inventory of Peer Influence on Children’s Eating and Body Concern, a self-reported tool created by Oliver and Thelen^(24)^, was utilised to evaluate the influence of peers on the eating habits of adolescent girls and, indirectly, on their nutrient intake. The tool includes three constructs: messages (frequency of negative body or eating habit messages), interactions (frequency of discussions, exercises or comparisons related to eating and body issues) and likability (belief that changing body weight or shape would improve peer or boy acceptance). Each construct had five-point Likert scale items, ranging from 1 (‘never’) to 5 (‘every day’) over a week. A total of eight items were used to measure these constructs, and scores were summed, with a possible range of 8–40 points. The mean score was calculated, and girls with a score above the mean were considered to have high peer pressure influence, while those with a lower score had low peer pressure influence^(25)^.

##### Body image perception

Body image perception was evaluated using a five-point Likert scale adapted from a study on body image perception in university students^(26)^. Adolescent girls were asked, ‘In your opinion, are you…’ with response options including ‘Far too thin’, ‘A little too thin’, ‘Just right’, ‘A little overweight’ and ‘Very overweight’. They were also asked whether they were satisfied with their current body weight or shape (yes/no). For analysis, the five responses were categorised into three groups: ‘Too thin’, ‘Just right’ and ‘Too fat’.

##### Food insecurity

Food insecurity was measured by the Household Food Insecurity Access Scale, which has a nine-item scale consisting of an occurrence question followed by a frequency of occurrence question during the previous month, which is a structured, standardised and validated tool developed by the United States Agency for International Development-funded Food and Nutrition Technical Assistance project. The participant’s response indicated a frequency of occurrence of never, rarely (1–2 times), sometimes (3–10 times) and often (> 10 times) for each of the questions, over the previous 4 weeks. Households that experienced none of the food insecurity (access) conditions, or just rarely experienced worry, were considered food-secure; otherwise, they were considered food-insecure^(27)^.

##### Mass media exposure

Respondents were asked about the frequency of their newspaper reading, radio listening or television watching. Those who reported engaging with any of these media at least once a week were considered to have regular exposure to that form of media^(20)^.

##### Meal frequency

The frequency of meals was obtained by asking the participants to identify the meals they usually had as breakfast, morning snack, lunch, afternoon snack, dinner and evening snack. Foods eaten between 07.00–09.00, 11.30–14.00 and 18.30–21.30 were regarded as breakfast, lunch and dinner, respectively, otherwise considered as snacks.

### Data quality control

The data collection tool (questionnaire) was initially prepared in English, then translated into the local language (Amharic) for data gathering and subsequently retranslated into English to ensure consistency. Data collectors received 2 d of training on the study’s objectives, the data collection process, how to use photographs of utensils for portion size estimation and proper interaction with participants. A pretest of the questionnaire was conducted with 5 %^(21)^ of the adolescent girls in the study area. The data collection process was supervised by a supervisor and the principal investigator, with daily checks for completeness. To maintain validity, the food portion weighing scale was recalibrated to zero after each measurement.

### Data management and analysis

The nutrient values per 100 g of each food item were mainly sourced from the Ethiopian food composition tables^(28,29)^. To determine the nutrient content of purchased foods, the nutritional information on their labels was used for analysis. These data were then input into Elizabeth Stewart Hands and Associates FOOD PROCESSOR software version 8·1 to build a nutrient database. The portion sizes of food items, obtained from a multiple-pass 24-h dietary recall, were manually converted into corresponding weights in grams. Subsequently, the daily intake values in grams were entered into the nutrient database. The software then computed the nutrient values for the portions of each food consumed by each individual. The results were transferred to Excel and exported to the Statistical Package for Social Sciences (SPSS) for further analysis.

Finally, energy and macronutrient intakes (carbohydrates, protein and fat) were compared with dietary reference nutrient intake set by WHO/FAO and IOM^(4,5)^. Adequate energy and protein intake was declared when the adolescent girls had an intake greater than or equal to RDA, whereas ‘inadequate intake’ was defined as intake lower than RDA. The collected data from other sections of the questionnaire (independent variables) were coded and entered into EpiData version 3.1 and exported to SPSS version 23. The data were sorted, cleaned and analysed using SPSS version 23. To determine the knowledge of participants on nutrients, first, adolescent girls who answered the knowledge assessment questions correctly were given a score of 1, and a score of 0 was given for those who did not correctly answer the question. After that, the total score of the correct answer and the median values of the knowledge score were calculated. Principal component analysis was used to determine the wealth status of respondents. The responses of all variables were classified into two scores. The highest score was coded as 1, and the lowest score was given the code 0. The assumptions of principal component analysis were checked to carry out the wealth index score. In principal component analysis, to determine the number of components that would be retained, the eigenvalue-one criterion was used, and those variables having a commonality value of greater than 0·5 were used to produce factor scores. Then, the score for each household on the first principal component was retained to create the wealth score. Finally, tertiles of the wealth score were created to categorise households as poor, medium and rich.

Descriptive statistics using mean (sd) for normally distributed, median (interquartile range) for non-normally distributed, frequencies and proportions were used to summarise variables. A bivariable logistic regression model was fitted. Variables with a *P*-value less than 0·2 were included in multivariable logistic regression analysis with backward elimination. The Hosmer–Lemeshow test was performed for model fitness in the final model. Having a *P*-value less than 0·05 in multivariable logistic regression analysis was used to conclude the presence of a statistically significant association. The strength of statistical association was measured by an adjusted OR at a 95 % confidence level. Finally, the results were presented in terms of text, frequency tables and graphs.

## Results

## Socio-demographic/economic characteristics of the respondents

A total of 401 adolescent girls were included in the analysis with a response rate of 95·02 %. Approximately two-thirds of 258 (64·3 %) of adolescent girls were in late adolescence (15–19 years). Two hundred forty-nine (62·1 %) respondents lived in rural settlements, and 349 (87 %) were Orthodox Christian religion followers. Substantial proportions – 363 (90·5 %) – of adolescent girls’ mothers did not have formal education (Table 1).

**Table 1:** Socio-demographic/economic characteristics of school adolescent girls at Meshenti Town, Bahir Dar City Administration, Northwest Ethiopia, February 2020 (*n* 401)

Variables	Frequency (*n*)	Percentage (%)
Age		
Early adolescent (10–14 years)	143	35·7
Late adolescent (15–19 years)	258	64·3
Residence		
Rural	249	62·1
Semi-urban	152	37·9
Religion		
Orthodox Christian	349	87·0
Muslim	52	13·0
Grade level		
Primary (4–8)	239	59·6
Secondary (9–12)	162	40·4
Mother educational status		
No formal education	363	90·5
Formal education	38	9·5
Father educational status (*n* 389)		
No formal education	306	78·7
Formal education	83	21·3
Mother occupation		
Housewife	333	83·0
Farmer	18	4·5
Merchant	31	7·7
Government employee	11	2·7
Others (daily labourer, student)	8	2
Father occupation (*n* 389)		
Farmer	261	67·1
Merchant	87	22·4
Government employee	32	8·2
Daily labourer	9	2·3
Family size		
≥ 5	308	76·8
< 5	93	23·2
Wealth index		
Poor	135	33·7
Medium	138	34·4
Rich	128	31·9
Household food security status		
Secured	279	69·6
In-secured	122	30·4

### Psycho-social and knowledge-related characteristics

About 92 (22·9 %) adolescent girls were not happy with their current body size. Generally, 236 (58·9 %) adolescent girls had an insufficient level of knowledge (Table 2).

**Table 2:** Social, personal and knowledge-related characteristics of school adolescent girls at Meshenti Town, Bahir Dar City Administration, Northwest Ethiopia, February 2020 (*n* 401)

Variables	Frequency (*n*)	Percentage (%)
Mass media exposure		
Non-exposed	224	55·9
Exposed	177	44·1
Peer pressure on eating habits		
High influence	186	46·4
Low influence	215	53·6
Body image perception		
Too thin	77	19·2
Just right	261	65·1
Too fat	63	15·7
Happy with current body weight		
Yes	309	77·1
No	92	22·9
Knowledge on nutrients		
Insufficient knowledge	236	58·9
Sufficient knowledge	165	41·1

### Energy and macronutrient intake of adolescent girls

More than 50 %, 57·6 % (95 % CI: 52·9 %, 62·8 %) and 18·5 % (95 % CI: 14·7 %, 22·2 %) of adolescent girls had inadequate intake for energy and protein, respectively. Only 1 (0·25 %) study participant had an inadequate intake of carbohydrates. Carbohydrate, protein and fat intake contributed to energy 75·22, 13·37 and 11·41 %, respectively. The median (interquartile range) of fat intake was 24·23 (18·24, 31·24). The median/mean intake of adolescent girls for energy, protein, carbohydrate and total fat was shown in Table 3.

**Table 3:** Mean (sd) and median (IQR) of macronutrients in school adolescent girls at Meshenti Town, Bahir Dar City Administration, Northwest Ethiopia, February 2020 (*n* 401)

Nutrient	RDA/RNI	Ref	MD	Mean	sd	Median	IQR (25th–75th)
Energy (kcal)	2200 kcal	WHO/FAO				2040·23	1648·24–2744·51
Protein (g)	34–46	WHO/IOM	13·37 %	63·88	20·99		
Carbohydrate (g)	130 (45–65 %)	WHO/IOM	75·22 %	359·39	101·39		
Fat (g)	25–35 %	WHO	11·41 %			24·23	18·24–31·24

RNI, reference nutrient intake; MD, macronutrient distribution for energy; IQR, interquartile range.

### Factors associated with energy and protein intake adequacy

### Associated factors of inadequate energy intake

In bivariable binary logistic regression analysis, adolescent girls’ religion, mothers’ education, dietary diversity, knowledge and meal eating frequency were factors for inadequate energy intake with *P*-value < 0·25 and were entered into multivariable analysis. Multivariable logistic regression analysis revealed dietary diversity, knowledge and meal eating frequency as the factors that were significantly associated with the energy intake adequacy of adolescent girls.

In this study, adolescent girls who had low dietary diversity were 4·31 times (AOR = 4·31, 95 % CI: (2·20, 8·47)) more likely to have inadequate energy intake compared with those who had high dietary diversity. The likelihood of having inadequate energy intake was 2·10 times higher among adolescent girls who had an insufficient level of knowledge (AOR = 2·10, 95 % CI: (1·34, 3·28) than those with a sufficient level of knowledge. Adolescent girls who consumed less than three meals per day were 2·51 times (AOR = 2·5, 95 % CI: (1·06, 5·95)) more likely to have inadequate energy intake than those who consumed three or more meals per day (Table 4).

**Table 4:** Factors associated with energy intake inadequacy among school adolescent girls at Meshenti Town, Bahir Dar City Administration, Northwest Ethiopia, February 2020 (*n* 401)

Variables	Energy intake adequacy				COR	95 % CI	AOR	95 %CI
	Inadequate		Adequate					
	*n*	%	*n*	%				
Religion								
Orthodox Christian	209	59·9 %	140	40·1 %	2·04	1·13, 3·67^*^		
Muslim	22	42·3 %	30	57·7 %	1·00			
Mother education								
No formal education	217	59·8 %	146	40·2 %	3·22	1·20, 8·67^*^		
Primary education	8	42·1 %	11	57·9 %	1·58	0·42, 5·95		
Secondary	6	31·6 %	13	68·4 %	1·00			
Dietary diversity								
Low	218	63·6 %	125	36·4 %	6·04	3·14, 11·62^*^	4·31	2·20, 8·47^*^
High	13	22·4 %	45	77·6 %	1·00		1·00	
Knowledge								
Insufficient	119	72·1 %	46	27·9 %	2·86	1·87, 4·38^*^	2·10	1·34, 3·28^*^
Sufficient	112	47·5 %	124	52·5 %	1·00		1·00	
Meal frequency								
< 3 meal	32	82·1 %	7	17·9 %	3·74	1·61, 8·71^*^	2·51	1·06, 5·95^*^
≥ 3 meal	199	55·0 %	163	45·0 %	1·00		1·00	

1:00 Reference group; COR, crude OR; AOR, adjusted OR.

* Significant at *P* < 0·05.

#### Associated factors of inadequate protein intake

In bivariable binary logistic regression analysis to determine factors associated with protein intake inadequacy class of students, family size, age of adolescent girls, place of residence, dietary diversity, meal frequency and knowledge were factors with *P*-value < 0·25 and were entered into multivariable analysis. Then, in multivariable logistic analysis, age of adolescent girls, place of residence, dietary diversity, meal frequency and knowledge were significantly associated factors with inadequate protein intake.

This study showed that early adolescent girls (10–14 years) were 1·89 times more likely to have inadequate protein intake than late-adolescent girls (15–19 years) (AOR = 1·89, 95 % CI: (1·08, 3·31)). In contrast to this, adolescent girls from rural areas were 73 % less likely to have inadequate protein intake as compared with those from urban areas (AOR = 0·27, 95 % CI: (0·15, 0·48)). Adolescent girls who had low DDS were 3·28 times more likely to have inadequate protein intake compared with those who had high DDS (AOR = 3·28, 95 % CI: (1·08, 9·98)). The odds of inadequate protein intake were 2·94 times higher among respondents who ate less than three meals per day than those who ate three or more meals per day (AOR = 2·94, 95 % CI: (1·35, 6·37)). Moreover, adolescent girls with an insufficient level of knowledge were 1·82 times more likely to have inadequate protein intake as compared with those with a sufficient level of knowledge (AOR = 1·82, 95 % CI: (1·04, 3·19)) (Table 5).

**Table 5:** Factors associated with protein intake inadequacy among school adolescent girls at Meshenti Town, Bahir Dar City Administration, Northwest Ethiopia, February 2020 (*n* 401)

Variables	Protein intake adequacy				COR	95 % CI	AOR	95 % CI
	Inadequate		Adequate					
	*n*	%	*n*	%				
Age								
10–14 years	41	28·7 %	102	71·3 %	2·74	1·64, 4·59^*^	1·89	1·08, 3·31^*^
15–19 years	33	12·8 %	225	87·2 %	1·00		1·00	
Residence								
Rural	29	11·6 %	220	88·4 %	0·31	0·19, 0·53^*^	0·27	0·15, 0·48^*^
Semi-urban	45	29·6 %	107	70·4 %	1·00		1·00	
Class of students								
Primary (4–8)	55	23·0 %	184	77·0 %	2·25	1·28, 3·96^*^		
Secondary (9–12)	19	11·7 %	143	88·3 %	1·00			
Family size								
≥ 5	50	16·2 %	258	83·8 %	0·56	0·32, 0·97^*^		
< 5	24	25·8 %	69	74·2 %	1·00			
Dietary diversity								
Low	70	20·4 %	273	79·6 %	3·46	1·21, 9·88^*^	3·28	1·08, 9·98^*^
High	4	6·9 %	54	93·1 %	1·00		1·00	
Meal frequency								
< 3 meal	14	35·9 %	25	64·1 %	2·82	1·39, 5·74^*^	2·94	1·35, 6·37^*^
≥ 3 meal	60	16·6 %	302	83·4 %	1·00		1·00	
Knowledge								
Insufficient	43	26·1 %	122	73·9 %	2·33	1·40, 3·89^*^	1·82	1·04, 3·19^*^
Sufficient	31	13·1 %	205	86·9 %	1·00		1·00	

1:00 Reference group; COR, crude OR; AOR, adjusted OR.

* Significant at *P* < 0·05.

## Discussion

More than half – 57·6 % (95 % CI: 52·9 %, 62·8 %) – of adolescent girls had energy intake below energy requirements for girls in this age group, regardless of physical activity level. This was consistent with the findings of the study in India (60·42 %)^(30)^. However, the result is lower than previous studies in South Africa (89·1 %)^(31)^ and Northeastern Tanzania (83·0 %)^(32)^. The possible reason for the observed discrepancy might be due to the difference in the reference used. In South Africa, the energy intake of girls was compared with energy requirement based on physical activity level (estimated energy requirement), but in the case of the present study, the energy intake for girls was compared with the average Estimated Energy Requirement/RDA regardless of physical activity level^(4)^. Besides, the discrepancy might be due to differences in the study setting and seasonal variation. In the study area from November to February (study period), crops were harvested, more available and affordable in the market, which might affect household food availability and food intake. However, the adolescents’ tendency to misreport food intake might have contributed to the under/overestimation of energy intake^(33)^.

The present study revealed that mean carbohydrate intake (359·4 (sd 101·39) g) was above RDA by approximately 176·5 %, and less than 1 %, 0·2 % (95 % CI: 0, 0·7 %), of them had inadequate carbohydrate intake. This is consistent with study findings obtained in Southern Ethiopia (0 %)^(17)^ and Southern Africa (0 %)^(31)^. On the other hand, a higher prevalence of inadequate intake was reported in Gondar (2·8 %)^(16)^ and Tanzania (58·9–68 %) in 2012^(34)^. The possible reason of explanation for the difference might be due to high consumption of starchy foods, simple sugar foods (biscuit, candy and sugar cane, soft drinks) and sweaty beverages among adolescent girls in this study and difference in the study period, indicating a transition towards intake of foods high in sugar but low in essential nutrients in the study area, which might be a risk for chronic diseases. Moreover, the school environment might have an effect on the accessibility and affordability of simple sugar foods and sweet beverages in shops near the schools for students in the study area.

Despite the mean protein intake being above the RDA approximately by 42 %, in this study, 18 % (95 % CI: 14·7 %, 22·2 %) adolescent girls had inadequate protein intake. This finding is lower than the study findings reported in Southern Ethiopia (60·9 %)^(17)^, South Africa (25 %)^(31)^ and Tanzania (60·1 % in early and 81·3 % in late adolescents)^(34)^. These discrepancies may be due to differences in consumption of foods that are a high source of protein especially legumes by the present study participants. But this low prevalence of inadequate intake in this study doesn’t give a guarantee for adequacy of protein in the study area since the common sources for protein were plant-based, which are low in bioavailability and high-quality animal source proteins such as meat, fish and egg were less consumed^(35)^.

The WHO/FAO or IOM^(4,5)^ recommendation states that the contribution of macronutrients for total energy intake should be between 45 and 65 % for carbohydrates, 10–30 % for proteins and 25–35 % for fats for the population of 4–18 years old. This study indicated that the contribution of macronutrients to total energy intake observed in adolescent girls was found above the acceptable limits set by the IOM for carbohydrates, and it was below the limit set for fat. However, it is in the acceptable range for protein. These findings are in line with the study reported in Southern Ethiopia, which revealed that carbohydrate intake was above (80 %) and fat intake (8 %) was below the acceptable limit set in adolescent girls^(17)^. In contrast, the study conducted in Brazilian adolescent girls revealed that the averages for the caloric contribution of carbohydrates (54·0 %), proteins (15·0 %) and lipids (31·0 %) were within the accepted macronutrient distribution range^(36)^.

The result of the adjusted analysis revealed that the likelihood of inadequate energy and protein intake increased in adolescent girls with insufficient knowledge about nutrients. This is in agreement with the study findings reported in the United Arab Emirates^(37)^ and a systematic review conducted in 2016^(13)^. This is because nutritional knowledge is a strong predictor of adolescent girls’ healthy eating behaviour and dietary intake^(13,37)^. This suggests that nutrition-specific educational interventions should be designed to build sound nutritional knowledge for adolescent girls.

In addition, this study demonstrated that DDS was negatively associated with inadequate energy and protein intake. The likelihood of inadequate energy and protein intake was higher among adolescent girls who had low DDS compared with those who had high DDS. This is supported by the study done on DDS as a measure of nutritional adequacy^(38)^ that reported a significant positive correlation between dietary diversity and nutrient adequacy. Similarly, a study in Nigeria indicated that dietary diversity enhances adequate intakes of food calories and proteins^(39)^. This might be because dietary diversity is the proxy indicator of diet quality or dietary habit^(22)^, and thus having poor dietary habits can lead to inadequate intake of nutrients including energy and protein. Besides, there is always a marked variability in the concentration of nutrients in a single food group. So, when the variety increases, nutrient content also increases since a single food group does not contain all nutrients in the right proportion. This suggests that only a mixed or diversified diet can satisfy the protein and energy requirements of adolescent girls. However, the DDS of adolescent girls was very low. In this study, only 14·5 % of adolescent girls had high dietary diversity. This might be due to the impact of the low socioeconomic status of adolescent girls’ families for accessing nutritious foods like animal sources of food, fruits and vegetables, which are usually high in cost, poor nutritional knowledge, behavioural factors like body perception and meal time skipping. Moreover, the types of foods available at the household and market level or in the school environment will also determine dietary diversity. In this study area, cereals are commonly produced at the agricultural level and are easily accessible at the household and market levels. In addition, unhealthy food types like candy, biscuits and sweet beverages are easily accessible and affordable around the school environment. But fruits, vegetables and animal sources of food are less accessible at the household and market level or the school environment. As a result, the dietary practice of study participants in the study area mainly depends on cereals, legumes and simple sugar-type foods.

Meal eating frequency was another factor that was negatively associated with inadequate energy and protein intake of adolescent girls. Those who ate less than three meals per day were more likely to have inadequate energy and protein intake than those who ate three or more meals per day. This is consistent with the systematic review report done in 2015 for energy intake^(40)^, but another systematic review in the USA reported an inverse relation between meal eating frequency and adequate protein intake^(41)^. The observed discrepancy might be due to a difference in the classification of meal frequency. In this study, meal frequency was classified as less than three and three or more meals, but a systematic review classified it as less than four and four or more meals.

Inadequate protein intake was 1·89 times higher among early adolescent girls than late-adolescent girls. This might be due to early adolescent girls spending more time out of home away from caregiver supervision and might be prone to consume less nutritious food and might skip a meal as a reason for playing. The other possible explanation might be due to cultural bias of favouring older age in food serving including nutritious ones in rural areas. However, the study findings reported in Bangladesh revealed that there was no significant difference in protein intake among different age groups^(42)^. Furthermore, the odds of inadequate protein intake were higher among adolescent girls living in urban areas than in rural areas. This could be explained by as more protein source crops were produced in rural areas, which might increase the household food availability and dietary intake.

Using a 24-h multiple-pass recall, using photographs of equipment and a list of staple foods and probing minimises recall bias in this study. Despite these methodological strengths, the study is not free from recall bias, social desirability bias and under- or overestimation of intake. A single 24-h recall might not take into account the day-to-day intake variation. To overcome this limitation, the maximum sample size and avoidance of data collection on holidays, special occasion days or weekends were used in this study.

The dietary reference intake values used in this study might not be fully representative of this study group though the international recommendation for developing countries like Ethiopia was used.

### Conclusions

This study revealed that energy and protein inadequacy was high among adolescent girls in the study area. Carbohydrate intake was above the acceptable macronutrient distribution range for energy. However, fat and protein intake were below and within the acceptable macronutrient distribution range, respectively. Moreover, dietary diversity, knowledge on nutrients and meal eating frequency were significantly associated with both inadequate energy and protein intake. Besides, being in the early adolescent age group and urban residence were identified as significant factors for inadequate protein intake in adolescent girls. Therefore, attention has to be paid to early adolescent girls and urban residents. Cultivation of crops, fruits and vegetables and rearing of small animals at the household level to improve dietary diversity and school feeding programmes to improve meal eating frequency are recommended. Strengthening the incorporation of nutrition education in the school curriculum should also be considered. Moreover, further analytical studies to identify other unexplored factors for inadequate protein and energy intake including out-of-school adolescent girls are recommended.
